# Fox Insight at 5 years - a cohort of 54,000 participants contributing longitudinal patient-reported outcome, genetic, and microbiome data relating to Parkinson’s disease

**DOI:** 10.1038/s41597-024-03407-9

**Published:** 2024-06-12

**Authors:** Joshua Gottesman, Yasir Karim, Jodie Forbes, Leslie Kirsch, Keaton Stagman, Monica Korell, Caroline Tanner

**Affiliations:** 1 The Michael J. Fox Foundation for Parkinson’s Research, New York, USA; 2 23andMe, Inc., Sunnyvale, USA; 3 University of California, San Francisco, USA

**Keywords:** Parkinson's disease, Genetic databases, Outcomes research, Parkinson's disease, Genetics research

## Abstract

Fox Insight is an online, longitudinal study of over 54,000 people with and without Parkinson’s disease, facilitating discovery, validation, and reproducibility in Parkinson’s disease research. The study administers routine longitudinal assessments, one-time questionnaires on an array of topics such as environmental exposure or COVID-19, plus genetic and microbiome data collection. Researchers can explore and download patient-reported outcomes data and Parkinson’s disease related genetic variants upon completing a Data Use Agreement. The full genetic data set, including approximately 650,000 single nucleotide polymorphisms for over 10,000 participants, and the microbiome data set for over 650 participants, can be requested with a heightened level of access. Since the first Fox Insight data descriptor was published in 2020, the data captured has been extended significantly, so this paper supersedes the previous one. Since then, the number of participants has increased by more than 20,000; an additional 1,747,729 surveys were completed; 130 gigabytes of genetic data were released; responses from 16 new one-time surveys were collected; and, data from one additional sub-study was made available.

## Background & Summary

Parkinson’s disease (PD) is one of the most common neurodegenerative diseases, with prevalence expected to increase over time^1,2^. PD is highly heterogeneous in terms of symptoms (both motor and non-motor), response to medication, and rate of progression among those affected. This variability has introduced challenges in understanding disease progression, clarifying underlying pathophysiology, providing meaningful treatments, and fully grasping which symptoms are most detrimental to patients. In-person trials classically enroll participants who already have access to specialist care, with milder symptomatology, better cognition, and less diversity than the general population^3,4^. As a result, remote observational studies, while not completely free from selection bias allow for larger sample sizes, longer observational periods, and deep experiential patient perspectives that are needed to improve our disease understanding.

Online data collection offers a mechanism to address these research challenges and has been effectively employed in other settings to achieve large sample sizes and facilitate data access and analysis, such as in the National Institute of Health’s *All of Us* Research Program^5^. Online surveys may pose less subject burden, and web-based recruitment can help ameliorate recruitment barriers for hard-to-reach populations^6^. Mobile technology, in particular, has helped to support a narrowing of the digital divide across several racial, ethnic, geographic, and age groups^7–9^. Internet usage among those over 65, the population most likely to develop Parkinson’s disease, has risen by 63 percentage points in the last two decades, with 75% reporting regular internet usage^10^. The rising ubiquity of internet access and usage, coupled with the burgeoning field of online research and enthusiasm towards developing and validating digital endpoints, creates a powerful opportunity to advance PD research through online data collection.

In addition, genetic variation is thought to play a significant role in Parkinson’s disease etiology likely in concert with environmental exposure^11^. In a minority of cases, a rare single gene variant is strongly associated with Parkinson’s disease. Other variants increase risk but have lower penetrance^12^. Multiple genetic variants have been aggregated into a genetic risk score and combined with phenotypic characteristics to classify people with or without Parkinson’s disease^13^. Remotely assessed self-reported genotype and phenotype information suggested different clinical subtypes in one online study^14^. Genetic variation and risk alleles are an important component to understanding many aspects of Parkinson’s disease, and genetic data is a large asset.

Fox Insight is an online study consisting of regularly administered questionnaires collected longitudinally that were initiated in 2017, the data from which can be used to improve understanding of participant lived experience^15^. Study eligibility is open to participants with and without self-reported PD. For those that do not self-report a diagnosis of PD, any connection to PD (e.g., relative, spouse, and/or caregiver) is captured to further characterize participant experience. Other potential risk factors or connections to PD such as environmental exposures and/or genetic variations are also captured. Given that the progression of PD can lead to challenges in motor and executive functions, the online platform also allows data entry to be deputized to someone in the PD participant’s circle of care, such as a partner/spouse or caregiver, helping to foster long-term participant engagement. The relationship of the deputized informant to the person with PD is specified, and all data entered by the informant is marked as such.

Fox Insight utilizes validated PRO instruments and PD-related questionnaires through the online platform. The surveys that a participant is assigned in each study visit are dependent on self-reported diagnosis. Longitudinally administered instruments are assigned to participants on either a quarterly, biannual, or annual basis. The cadence with which instruments are administered are aimed at enabling investigators to understand change in a participant’s lived experience over time and/or understand a time-specific issue (e.g., COVID-19)^16^. Though the reliability of self-reported diagnosis relies on the accuracy of the information provided by participants, previous and ongoing studies have found high concordance rates between self-report and clinician-determined diagnosis^14,17^. Fox Insight also includes the implementation of one-time questionnaires, microbiome, and genetic data collection^15^. By design, Fox Insight can support modifications to multi-modal data collection in alignment with evolutions in Parkinson’s disease research. This flexibility is enabled by Fox Insight’s infrastructure, an agile-developed web application, built through a software development framework that emphasizes phased deployment, that manages enrollment, e-consent, collection of routine longitudinal assessments, and integration with external data capture systems such as Qualtrics to rapidly collect data from participants on timely research questions^18^.

For an abridged list of recent publications utilizing data generated by Fox Insight, please visit: https://foxden.michaeljfox.org/insight/explore/publications.jsp.

## Methods

Fox Insight is open to participants from any country, aged 18 or older, who provide informed consent through the Fox Insight website; informed consent and study protocol are reviewed by the WCG IRB (IRB#: 120160179, Legacy IRB#: 14–236, Sponsor Protocol Number: 1, Study Title: Fox Insight). A data descriptor for Fox Insight was first published in 2020, but since then the data captured has been extended significantly, so this data descriptor supersedes the previous one^19^.

Upon registration into Fox Insight, participants are divided into two primary cohorts, those with Parkinson’s disease and those without. Once divided, participants are given a different set of assessments based on self-reported Parkinson’s disease diagnosis. Importantly, participants without PD are asked about new diagnoses every three months, and participants with PD are asked whether their diagnosis has been changed by a health care professional since their last Fox Insight visit.

People with Parkinson’s disease respond to health, non-motor assessments, motor assessments, quality of life, and lifestyle questionnaires. Over the course of a year, people with PD are assigned 54 questionnaires with an average of 13.5 questions per assessment. On the other hand, people without PD are assigned 37 questionnaires over the same period, with an average of 9.25 questions per assessment. The number of questionnaires assigned to participants for the same “visit” may vary. This is due to several factors such as the removal or addition of a questionnaire or assignment cadence of a questionnaire changing.

Prior to September 2019 participants had a 90-day window to complete all questionnaires assigned during a longitudinal assessment. There was no buffer period between assessment periods and participants could potentially complete two longitudinal assessments in two consecutive days. After September 2019, participants have 30 days to complete each assessment, and there is a 60-day buffer period between routine longitudinal assessments.

Participants that meet the pre-set eligibility criteria of optional, one-time questionnaires are invited to participate in additional PRO collection. The eligibility criteria for these additional questionnaires can vary based on the survey topic. Invitations to one-time questionnaires may only be segmented by reported PD diagnosis; any additional inclusion/exclusion criteria are applied via questionnaire skip logic. Participants may also be eligible to join sub-studies to contribute other types of data, such as genetic and microbiome records.

During the first six months following the study’s beta period (for more, see the *Beta Participants* section), there were 2,868 participants who did not complete demographic questions in About You; a subset of approximately 500 individuals skipped this questionnaire due to a platform glitch which was resolved in Q3 2017. There are 1,476 participants who had two consecutive assessment periods starting on the same day (i.e., questionnaire responses are associated with the same “Days since Acquired” variable). This questionnaire assignment error has since been fixed. The resulting output for these participants includes data from the most recent, later, routine longitudinal assessment; data from former assessments have been skipped.

Fox Insight has leveraged advancements in mobile technology. Online surveys and web-based recruitment allowed Fox Insight to maintain stable participation during the COVID-19 pandemic. Participants completed 46,892 longitudinal assessments during the 12-month period leading up to March 2022, and completed 43,835 longitudinal assessments in the subsequent 12 months. Furthermore, 10,000 participants, nearly one-fifth of the entire cohort, have joined the study since March 2020.

**Fig. 1:**
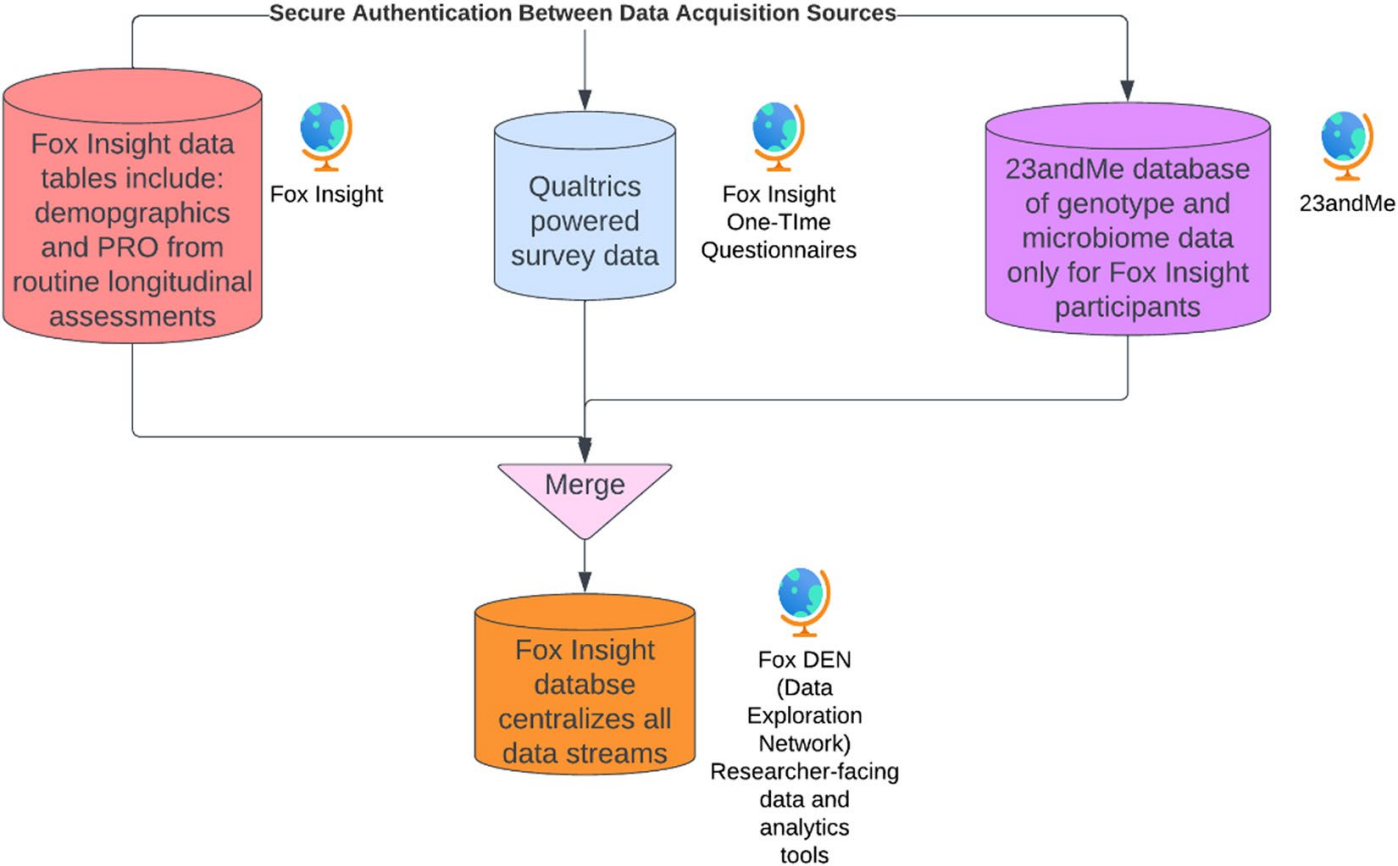
Fox Insight Data Flow.

Figure 1 below represents the data flow in Fox Insight combining patient-reported outcomes, genetic, and microbiome data into Fox Insight’s data ecosystem. Demographic data and patient-reported outcomes from routine longitudinal assessments are merged with responses from one-time questionnaires, microbiome, and genetic data into a central database accessible to researchers.

The following method describes the three data acquisition sources of Fox Insight: routine longitudinal assessments, one-time questionnaires, microbiome, and genetics as illustrated in Fig. 1. Routine longitudinal assessments form the main study activities and are collected through a custom survey application developed by Mondo Robot, a creative digital agency. One-time questionnaires are deployed through Qualtrics® survey software, leveraged for additional survey programming rules. Finally, genetic and microbiome data are collected in collaboration with 23andMe, Inc., a personal genetics company.

### Routine longitudinal assessments

Routine longitudinal assessments are offered to participants every three months and are hosted through an online survey platform. Questionnaires are assigned to participants based on their self-reported Parkinson’s diagnosis at the beginning of the assessment. These assessments aim to comprehensively evaluate the different dimensions of Parkinson’s disease. When possible, validated instruments are used such as the Movement Disorders Society – Unified Parkinson’s disease Rating Scale (MDS-UPDRS) Part II. Data collection from routine longitudinal assessments is governed by survey logic. For more details, please refer to the previous version of this paper authored by Luba Smolensky *et al*.^19^.

### One-time questionnaires

One-time questionnaires (Tables 1–3) are deployed through Fox Insight to enrich the PRO data collected through routine longitudinal assessments with additional validated instruments^15^. These questionnaires can collect cross-sectional data from novel or unique instruments not included in routine longitudinal assessments. For instance, one-time questionnaires can obtain participant perspective during research development, evaluate interest in specific interventions, target recruitment for clinical trials, or ask time-sensitive questions pertaining to topics such as the impact of the COVID-19 pandemic. The ability to deploy one-time questionnaires is an enormous advantage of the Fox Insight platform. The frequency and content of questionnaires is vetted by study leadership to ensure alignment with Fox Insight’s scientific goals; instruments must be IRB-approved before deployment.

**Table 1:** One-Time Questionnaires Deployed in Fox Insight as at 03-01-2023 to the PD Cohort only.

Survey Topic	Deployment Period	Responses	Participating Cohort
Assessing Discrimination in Healthcare*	June 2021 - January 2023	5,367	All participants with PD
Cannabis use in PD*	January 2020 - January 2023	5,618	All participants with PD
Compensation Strategies to Improve Walking in Persons with PD*	March 2020 - January 2023	8,131	All participants with PD
Impact and Communication About OFF Periods	February 2018 - March 2018	1,290	All participants with PD
Patient Therapeutic Preferences Questionnaire by the Medical Device Innovation Consortium	November 2017 - January 2018	4,667	All participants with PD
The Role of Stress in Parkinson’s Disease*	August 2019 - September 2019	5,036	All participants with PD
Understanding Fatigue in Parkinson’s Patients*	March 2019 - June 2019	1,647	All participants with PD

*asterisk indicates questionnaires new since the 2020 version of the data descriptor.

**Table 2:** One-Time Questionnaires Deployed in Fox Insight as at 03-01-2023 to All Participants.

Survey Topic	Deployment Period	Responses	Participating Cohort
Attitudes and Beliefs Regarding Research and Genetic Testing for PD*	January 2022 - January 2023	6,512	All participants
COVID-19 Experience in the PD Community:			
Part-1*	April 2020 - March 2021	9,146	All participants
Part-2*	June 2021 - January 2023	7,619	
Environmental Exposure Questionnaires (PD-RFQ-U):			
*Alcohol*	October 2017 - March 2019	2,830	All participants
*Caffeine*		6,495	
*Smoking and Tobacco*		3,833	
*Head Injury and Concussion*		3,106	
*Pesticides at Work*		3,070	
*Pesticides in Non-Work Settings*		2,805	
*Residential History*		2,792	
*Physical Activity and Sleep*		3,615	
*Height and Weight*		3,746	
*Calcium Channel Blocker Medication History*		2,926	
*Anti-Inflammatory Medication History*		1,988	
*Occupation*	October 2017 - January 2020	6,124	
*Toxicant*	October 2017 - February 2019	3,066	
*Female Health History*	October 2017 - December 2019	4,770	
Experience with Sensory Misperceptions*	July 2020 - January 2023	8,447	All participants
Medication Deprescribing & Clinical Research Study Participation*	March 2022 - March 2023	6,901	All participants
Mood disorders and Parkinson’s disease*	July 2021 - January 2023	8,120	All participants
Repetitive Head Impact*	November 2020 - January 2023	7,659	All participants

*asterisk indicates questionnaires new since the 2020 version of the data descriptor.

**Table 3:** One-Time Questionnaires Deployed in Fox Insight as at 03-01-2023 to specific sub-cohorts.

Survey Topic	Deployment Period	Responses	Participating Cohort
Care Partner Experiences*	November 2021 - January 2022 and May 2022 - present	2,751	All participants without PD (care partners)
The Financial and Social Impact of Parkinson’s disease Survey	September 2018 - July 2019	1,846	All US-based participants
The Role of Stress in Parkinson’s Disease (Control)*	October - December 2019	1,292	All participants without PD
Understanding Psychosis and Its Burden on Caregiver*	September - October 2019	740	All Participants without PD (caregivers)
Experiences of Women Living with PD: Female Health and Home Life*	May 2022 - present	3,464	Female participants with PD
Experiences of Women Living with PD - Pre, Peri, and Post Menopause*	January 2023 - present	2,219	Female participants with PD
Understanding OFF and ON in Parkinson’s disease Patients	November 2018 - December 2019	2,886	Participants with PD who report taking at least one PD-related medication

*asterisk indicates questionnaires new since the 2020 version of the data descriptor.

### Sub-studies

#### Fox Insight genetic data

In partnership with 23andMe, approximately 10,000 participants with Parkinson’s disease in the US were enrolled in the Fox Insight Genetic Sub-Study (FIGS) from 2017 to 2021. Participants were genotyped for around 650,000 single nucleotide polymorphisms (SNPs). Genotyping was performed on participants who had completed at least one routine longitudinal assessment, thereby ensuring that researchers are able to explore correlations between genetic variations and phenotypic manifestations. Eligible participants provided samples using 23andMe’s saliva collection kit and these were genotyped on a variety of genotyping platforms. Within Fox Insight, 6.9% of participants were genotyped on the V3 platform which is based on the Illumina OmniExpress + BeadChip and contains a total of about 950,000 SNPs, 12.7% of participants were genotyped on the V4 platform which is a fully custom array of about 570,000 SNPs, and 80.4% of participants were genotyped on the V5 platform which is in current use and is a customized Illumina Infinium Global Screening Array of about 650,000 SNPs. As part of the resulting dataset, several genetic variants that may be relevant for Parkinson’s disease research (including variants located near GBA, LRRK2, APOE, PRKN, MCCC1, BIN3, and the HLA loci) are available in tabular form alongside phenotypic data in Fox Insight’s public repository^15^. These variants are included as categorical data to democratize data access and interpretation for otherwise complex SNP output (the full set of SNPs is available upon request to qualified researchers, subject to completion of the Tier 2 data use agreement described in the Usage section of this paper). For additional information, researchers with access to these data may refer to the genetic platform annotations available on the resources section of Fox DEN: https://foxden.michaeljfox.org/insight/explore/insight.jsp?#fox-resources. The genetic platform annotations provide researchers who have access to the Tier 2 data with summary statistics covering genotyping rates by platform, as well as a brief overview of the different platforms themselves, including the estimated number of SNPs per array.

#### Fox insight microbiome data

In partnership with 23andMe, gut and oral microbiome data was collected from approximately 650 participants, both those with PD (approximately 65%) and those without PD (approximately 35%) as part of the Parkinson’s Disease Microbiome (PDMB) Sub-Study. The preponderance of PDMB participants were also FIGS participants. The study aimed to investigate the differences in the gut and oral microbiomes of individuals with PD compared to individuals without PD. Shallow shotgun sequencing reads from stool and saliva samples were collected and a one-time questionnaire about diet, medication use, and oral hygiene was completed. The study was conducted in two phases, with an initial pilot phase focused on testing the feasibility of collecting stool and saliva samples from participants in December 2019. The main phase of the study was conducted between February and December of 2021 with a wider group of participants. Responses to the one-time questionnaire are available in tabular form alongside phenotypic data in Fox Insight’s public repository^15^. The PDMB sequencing data set for both FIGS and non-FIGS participants is available upon request to qualified researchers.

For this sub-study, DNA extraction, sequencing, and annotation were performed by Diversigen (Minnesota, USA). Upon receipt of the samples (DNA Genotek’s OMR-200 stool kit and OM-501 saliva kit), visual observations of the collection kits were recorded, including sample discoloration, low volume, and incorrect usage. Additional quality controls (QC) included determining that there were at least 16,000 16 S copies/μL and greater than 1 ng/µL of DNA per sample. Microbial DNA was extracted at room temperature from saliva and stool samples using the QIAGEN Powersoil Pro DNA isolation kit with a liquid-handling robot, and according to standardized protocols (QIAGEN, Netherlands). Sequence libraries were prepared using enzymatic tagmentation with low cycle polymerase chain reaction. 88 samples plus a positive and negative library prep control were included in each 96 well-plate. Sequencing was performed with the BoosterShot® shallow shotgun sequencing platform, with 1 × 100 reads at a target depth of 2 million reads per sample. The Cutadapt software tool was used to discard sequences with an average Q score less than 30. Additionally, samples with fewer than 10,000 reads were discarded. For taxonomic annotations, the remaining quality reads were aligned to a database containing all representative genomes in NCBI’s RefSeq for bacteria with additional manually curated strains (Diversigen). Alignments were made at 97% identity against all reference genomes. Every input sequence was compared to every reference sequence in the Diversigen Venti database using fully gapped alignment with BURST. Ties were broken by minimizing the overall number of unique Operational Taxonomic Units (OTUs). For taxonomy assignment, each input sequence was assigned the lowest common ancestor that was consistent across at least 80% of all reference sequences tied for best hit. For functional group annotation, Kyoto Encyclopedia of Genes and Genomes Orthology groups (KEGG) orthologies (KOs) were observed directly using alignment, via the Prokka software tool, at 97% identity against a gene database derived from the strain database used above (Venti). KOs were collapsed to level-2 and -3 KEGG pathways and KEGG Modules.

For both the genetic and microbiome sub-studies, 23andMe, Inc. provided analysis to study participants free of charge. Study participants provided informed consent permitting the company to use their data in a de-identified manner for research purposes.

#### AT-HOME PD

169 Fox Insight participants were enrolled in the Assessing Tele‐Health Outcomes in Multiyear Extensions of Parkinson’s Disease or the ‘AT-HOME PD’ (AHPD) sub-study. This remote observational study was funded by the National Institute of Neurological Disorders and Stroke and completed in collaboration with multiple institutions. Participants from two separate phase III clinical trials were invited to participate in AT-HOME PD: from STEADY-PD III and from SURE-PD 3^20^. AHPD had three main components: (1) annual video visits conducted from the University of Rochester, (2) smartphone based assessments and passive data collection using the mPower 2.0 Parkinson’s smartphone application by Sage Bionetworks, and (3) Fox Insight routine longitudinal assessments^21^. Data collected from the study will be transferred to the Parkinson’s Disease Biomarkers Program (PDBP) Data Management Resource and merged with the data from the parent clinical trials^22^. The study was initially funded and ran from October 2018 through April 2023. The trial was extended through 2028 – with visits expected to run through 2027 – in April 2023^23^. This extension, ‘AT-HOME PD2’, will help to evaluate the extent to which remote reporting can improve the prediction of important clinical milestones.

#### Telemedicine verification sub-study (FIVE)

203 Fox Insight participants were enrolled in the Telemedicine Verification (“FIVE”) sub-study. The purpose of this study was to validate self-reported Parkinson’s diagnosis in Fox Insight through a virtual visit. Research team members based at the University of Rochester conducted all virtual visits^24^. Participants were divided into five approximately equal cohorts; four cohorts of those with self-reported PD diagnosis of varying lengths, and one without PD. Participants completed a series of questionnaires before the video-based visits related to demographic information and PD diagnosis etc. During the virtual visits, the investigator reviewed medications, collected a health history, and performed a modified MDS-UPDRS motor examination among other tests. Questionnaire data collected from this study are available in tabular form alongside phenotypic data in Fox Insight’s public repository^15^.

#### Data centralization

Participant answers to routine longitudinal assessments, one-time questionnaires, microbiome, and genetic data are integrated in a public repository managed at the USC Laboratory of Neuro Imaging (LONI), Mark and Mary Stevens Neuroimaging and Informatics Institute. Using dates of birth provided during user registration, dates associated with participant answers are converted to participant ages to protect participant confidentiality. For more information on how time is represented in the Fox DEN database, researchers may visit: https://foxden.michaeljfox.org/insight/explore/time.jsp. This webpage provides an overview to help data users understand Fox Insight’s data related to participant age (along with how it is randomized), and how to interpret the temporal relationship between study observations.

As questions for a single routine longitudinal assessment may be edited and answered intermittently, the total number of days used to complete each survey is also recorded for each participant. Along with dates of birth, unrestricted and free form textual answers are quarantined from the general public data set; when appropriate, “derived” variables are defined for those questions to filter out arbitrary and participant-identifying responses. Derived variables are also added for cases in which participants are allowed to answer a question in different ways (e.g., enter weight in pounds or kilograms) in order to standardize these responses. For a detailed catalogue of variables (both derived and otherwise), researchers may download a copy of the data dictionary at: https://foxden.michaeljfox.org/insight/explore/insight.jsp.

## Data Records

Data collected from each survey is aggregated into a single table and is available via a comma separated value (CSV) file. Data is available via monthly data cuts containing all survey records at the end of each month. This paper uses data from the March 2023 data cut. Variable values are encoded according to a data dictionary, which accompanies each download of the data deposited in the Fox Insight Data Exploration Network (Fox DEN) repository at https://foxden.michaeljfox.org (access and usage notes detailed in later sections)^15^. From the Fox DEN user interface, users can select entire surveys for download, or select only variables of interest. Further, if a user is interested in variables from multiple surveys, there is the option to download these simultaneously, either separately (as collected), or automatically joined. Participant ages are provided alongside time-dependent data. Additional metrics (e.g., variable vectors per subject recording data availability, histograms of variable values) are pre-computed to facilitate searching and data grouping by researchers. The data dictionary (Table 4) describes metadata for the collected variables for each survey question in the routine longitudinal assessments and one-time questionnaires. The complete data dictionary in PDF format, of 7,036 collected variables (as at 03-01-2023), is available for download in Fox DEN^15^. This version of the data dictionary, includes accompanying introductory texts that describes the structure of the data (i.e., long format), and covers common topics like how to interpret diagnosis status and disease duration, while providing an overview of common tables of interest (e.g., pertaining to demographics) and frequent transformations (such as merging multiple tables).

**Table 4:** Sample extract of the Data Dictionary for Fox Insight Assessments detailing the “Care Partner Experiences” one-time survey.

Variable	Data Type	Question Text	Value	Value Description	Notes/Resources
CPEPDiag	Numeric	Do you currently have a diagnosis of Parkinson’s disease, or parkinsonism, by a physician or other health care professional?	1	Yes	Participants are skipped to the end of the survey if they didn’t respond with ‘Yes’ to this question.
			2	No	
CPERegActivity	Numeric	Consider only how much health problems affected your ability to do your regular daily activities, other than work at a job.	0	Scale of impact	Participants rated the effect of caregiving on a scale of 0–10.
			1		
					0 = Caregiving had no effect on my work.
			2		
					10 = Caregiving Completely prevented me from working.
			3		
			4		
			5		
			6		
			7		
			8		
			9		
			10		
CPEPrimary	Numeric	Do you identify yourself as a primary care partner for a person with Parkinson’s disease?	0	Not Checked	Participants are only presented this question if they answered, ‘Yes’ to the question in Variable ‘CPEUnpaid’. (CPEUnpaid = = 1)
			1	Checked	

## Technical Validation

Technical Validation for Fox Insight is bifurcated into tool and data validation. Data validation closely reviews caveats associated with collecting participant reported outcomes and compares the sex chromosome to self-reported sex for genetic data validation.

### Deployment of routine longitudinal assessments

To verify the appropriate deployment of routine longitudinal assessments, development tests are routinely conducted by Mondo Robot. While platform tests verify that questionnaires are deployed according to set intervals, post-tests spot check data collection nuances from said tools. For additional information, please refer to the prior publication on this topic (see Citation #20).

### Collected data

The data collection methods converge to form a large sample size of PROs from routine longitudinal assessments, one-time questionnaires, microbiome, and genetic data as illustrated^15^. Potential duplicate records are removed in upstream data management stages.

Table 5 details the scale of collected data in Fox Insight and key cohort characteristics. As of 03-01-23, there were over 38,000 people with Parkinson’s disease enrolled making Fox Insight the largest prospectively followed Parkinson’s disease cohort worldwide, exceeding the second largest cohort of 13K people with Parkinson’s disease followed in the Parkinson’s disease Outcome Project. Of the 54,190 total individuals enrolled in Fox Insight, 70.7% (n = 38,299) participants are people with Parkinson’s disease. The average age of the Parkinson’s disease cohort is 65.8 and these participants, by average, have had a PD diagnosis for more than 5 years.

**Table 5:** Demographics and Collected Data in Fox Insight (as of 03-01-2023).

Full Cohort (N = 53,993)	People with Parkinson’s disease	People without Parkinson’s disease
Total enrolled N (% of total)	38,299 (70.7%)	15,891 (29.3%)
Age* in mean years	65.8	56.1
Sex		
Female	16,068	11,120
Male	20,137	3,749
Length of Parkinson’s disease diagnosis* (mean years)	5.3	NA
Full Data Collection		
Completion in first routine longitudinal assessment:		
Participants who completed first assessment (% of cohort)	25,720 (67.2%)	9,986 (62.8%)
Questionnaires completed in first assessment	485,331	132,386
Completion in subsequent routine longitudinal assessments:		
Participants who completed at least two assessments (% of cohort)	20,742 (54.2%)	6,388 (40.2%)
Questionnaires completed in subsequent assessments	1,586,914	351,180
Assessments completed per participant (mean (sd))	3.82 (5.6)	2.68 (5.1)
One-Time Questionnaires Completion:		
Participants (% of cohort)	17,894 (46.7%)	5,019 (31.6%)
Questionnaires (average per participant)	115,834 (6.5)	25,667 (5.1)
Genetic Sub-Study:		
Total genotyped	10,710	NA

Note that some figures do not tally exactly due to missing values^15^.

Note: ‘Age’, ‘Sex’ and ‘Length of Parkinson’s Disease diagnosis’ are calculated from the time of Fox Insight registration. PRO data as of 03-01-2023. Enrollment in the Genetic Sub-study as of 03-01-2023.

As of 03-01-23, the Fox Insight dataset has a larger sample size available for cross-sectional analysis than longitudinal; 67.2% (n = 25,720) of people with Parkinson’s disease have completed the first routine longitudinal assessment and 54.2% (n = 20,742) of people with Parkinson’s disease participants have completed at least two routine longitudinal assessments^15^. As routine longitudinal assessments are completed sequentially, there is observed drop-off from the first to the last assessment within the same period of approximately 10%. Participants who miss 3 or more consecutive longitudinal assessments are more likely to remain dormant rather than return to complete further assessments. People without Parkinson’s disease exhibit a similar trend in assessment completion. Approximately 16,000 participants provided responses to questionnaires solely on the day of their registration.

Optional one-time questionnaires are completed by a comparatively lower proportion of the study population: 46.7% (n = 17,846) of participants with Parkinson’s disease, and 31.6% (n = 5,019) of participants without Parkinson’s disease completed at least a single one-time survey. Participants who completed multiple longitudinal assessments are more likely to complete these one-time questionnaires regardless of disease cohort. The number of respondents to an individual one-time survey varies considerably, from a low of 740 completing Understanding Psychosis and Its Burden on Caregiver to a high of 9,146 completing COVID-19 Experience in the PD Community – Part1. It is worth noting that the eligible population for each survey varies: in this case the former was limited to study participants caring for someone living with PD and the latter open to all study participants.

### Beta participants

Approximately 8% of total participants as of 03-01-23 were part of Fox Insight’s beta group, defined as those joining before the March 2017 soft launch of Fox Insight. Data collected during the beta period (defined as July 2014 to February 2017) could be subject to questionnaire versioning and inconsistencies associated with platform troubleshooting and optimization. Because of this, data collected during this time is not publicly available. However, data collected from participants who enrolled during this time but continued on to contribute data following March 2017 are publicly available^15^.

## Usage Notes

### Access

To access Fox Insight data through the Fox DEN tool, researchers are asked to complete and e-sign a data use agreement (DUA) at https://foxden.michaeljfox.org. The DUA governing Fox Insight data contains certain restrictions on use to align with specific assurance on data use and reuse provided to participants via the Informed Consent process. This includes a prohibition on data redistribution to ensure data governance is consistent with these assurances; Fox Insight’s data management core takes data returns as necessary from researchers to assist in redistribution of derived data and/or sub-study data to ensure these results are easily made available to the research community. The DUA also contains certain restrictions on intellectual property, which were determined to be the best legal solution to ensure that Fox Insight usage is consistent with explicit promises to participants that their data would be used for research purposes. These restrictions have been proven as a workable solution for thousands of Fox Insight data users over the five years that the dataset has been available for re-use.

There are two sets of data use agreements; the first (Tier 1) allows researchers to access responses from routine longitudinal assessments, one-time questionnaires, and 17 pre-selected Parkinson’s disease-related genetic variants. Separately, the second (Tier 2) data use agreement allows researchers, with institutional review, to request access to all SNPs and microbiome data. Data dictionaries and genetic data documentation are available in Fox DEN as reference guides.

Researchers can register for an account through Fox DEN and upon successful completion of the Fox Insight data use agreement, researchers can explore, analyze, and download data as illustrated. Users have the option to either download selected variables of their choice through the graphical user interface (see below) or access monthly data cuts via a static 10.25549/bxya-6133^15^. When referencing this DOI, researchers are asked to reference the corresponding Archive Data of the accession of data used in analysis.

### Fox DEN User interface

**Fig. 2:**
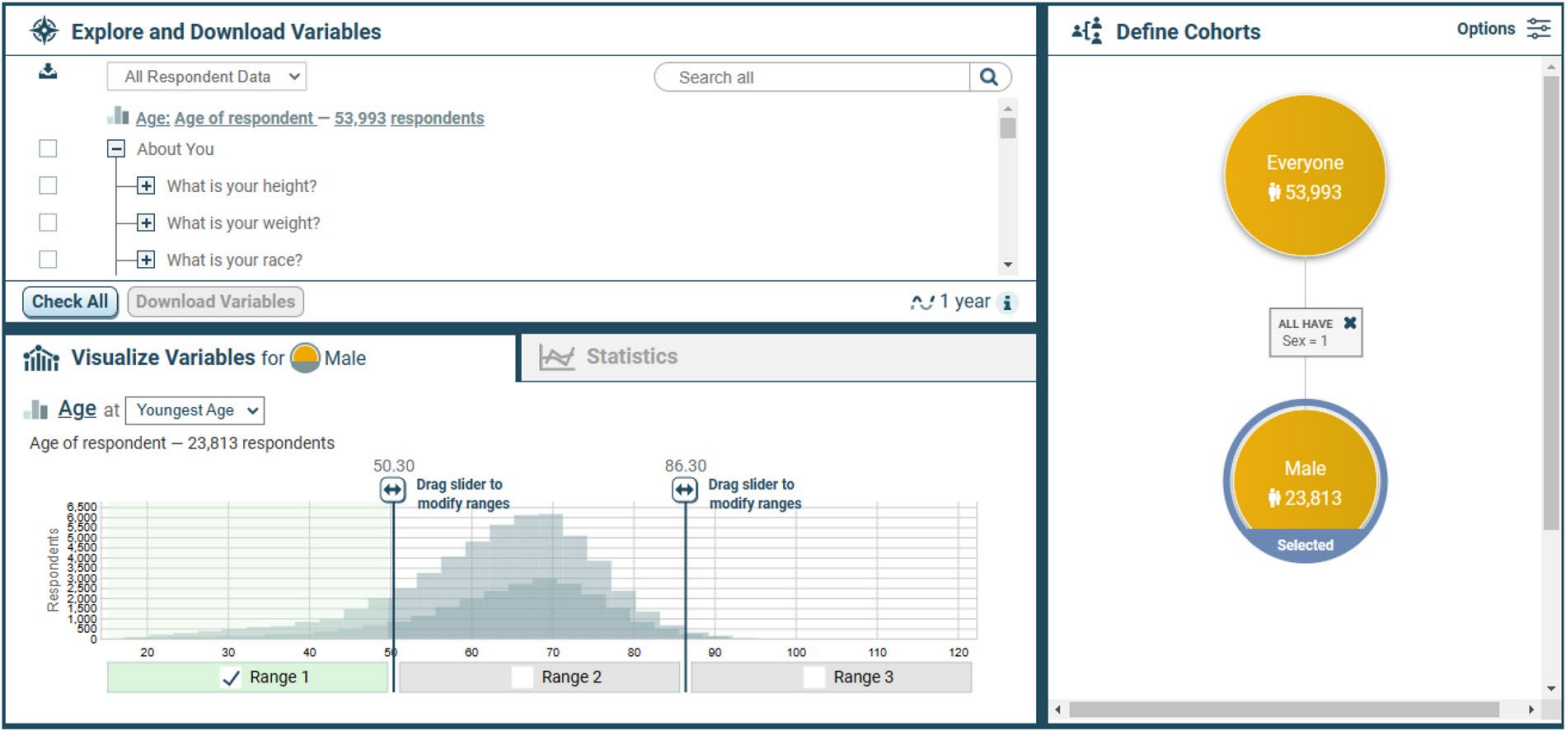
The Fox DEN user interface.

Using the Fox DEN interface (Fig. 2), investigators may explore, select data, and apply statistical methods using user-created cohorts based on subject demographics, PROs, and SNPs. Routine longitudinal assessments, one-time questionnaires, and genetic data are organized in a tree structure. The tree is filtered using drop-down categories (e.g., questionnaires, genetic data, sub-study data) or keyword searches^15^. The distributions of participants’ questionnaire responses and SNP variants are visualized when selected in the tree. Categorical variables can be reduced to user-defined binary variables, which are useful inputs to the statistical methods. Variable visualizations are dependent upon the user-selected cohort, and this provides visualizations specific to subsets of participants. Cohorts are created by recursively selecting values of a variable and using them as a filter to subset a parent cohort. Cohorts are viewed in a tree structure that shows how the cohorts are inherited from one another as well as the filters that define them. Fox DEN supports common statistical methods (linear correlation, logistic regression, chi-square and T-test) through drag and drop operations of its cohorts and variables. A “Guided Statistics” wizard provides step-by-step guidance in choosing appropriate statistical methods for user selections. While most (but not all) variables can be used to generate a range chart or bar chart for the purposes of quick exploration, it is recommended that researchers leverage the monthly data cuts (as described in the Data Records section) for researcher purposes.

**Fig. 3:**
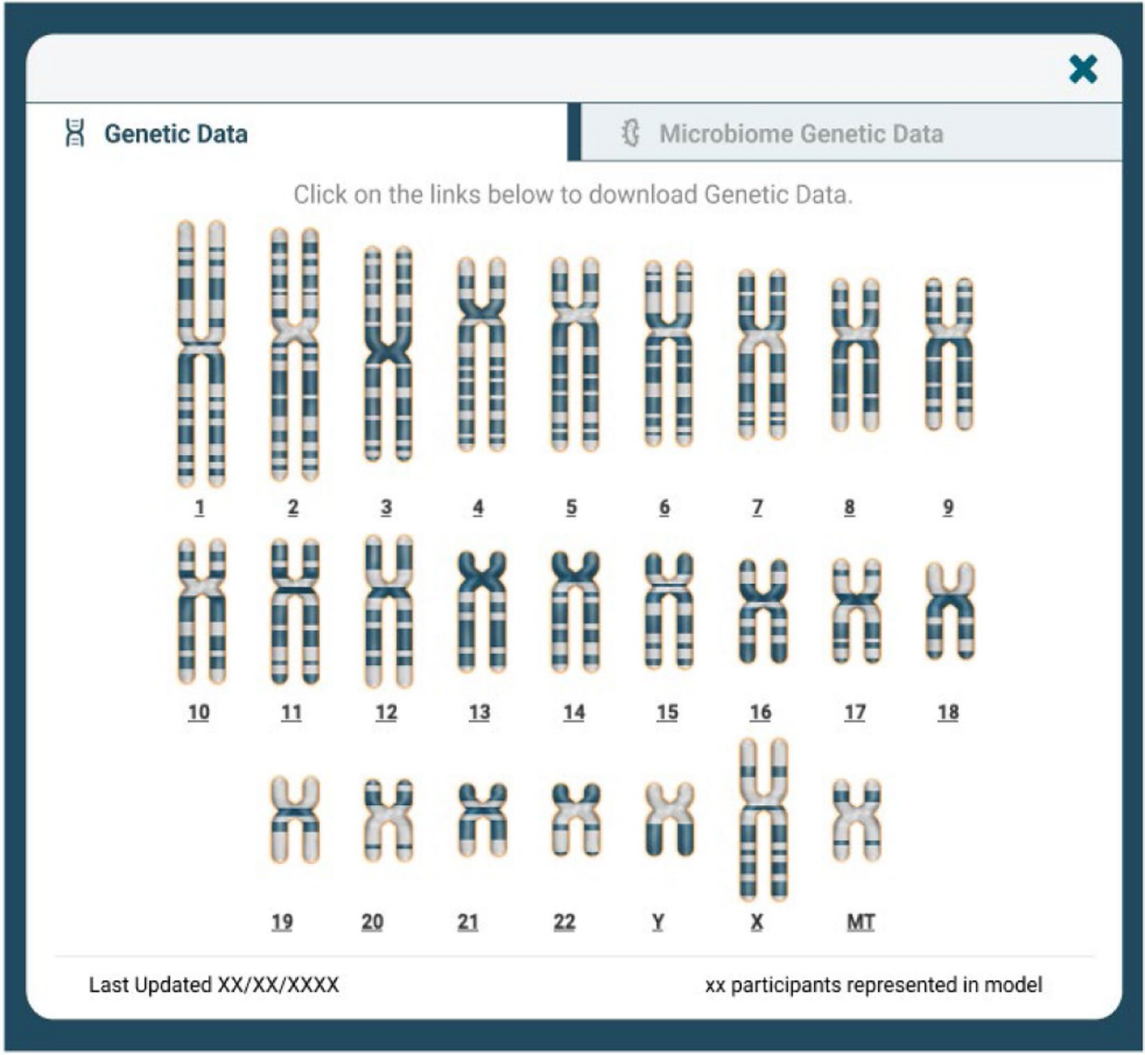
Interface for downloading genetic data in Fox DEN.

**Fig. 4:**
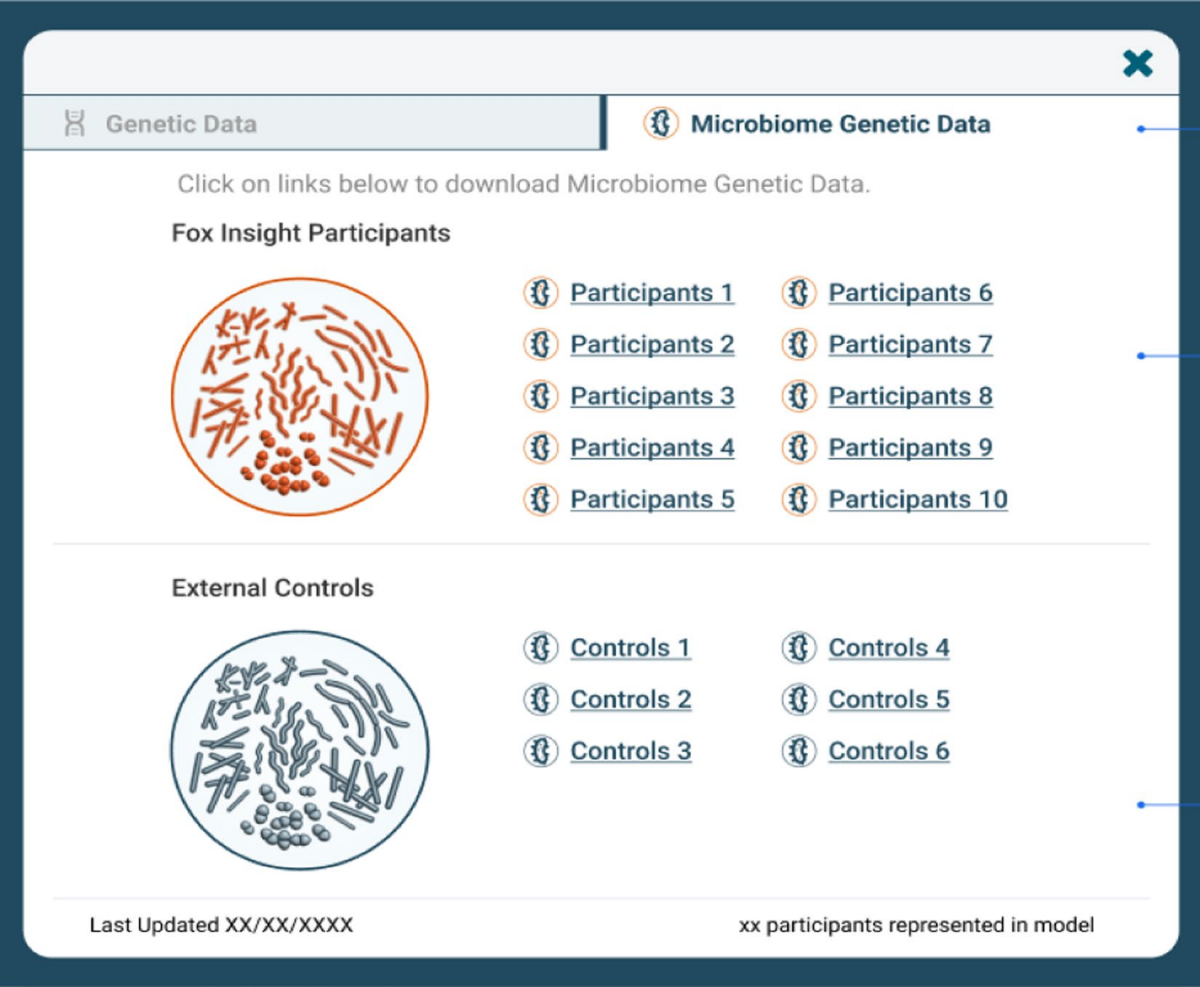
Interface for downloading microbiome data in Fox DEN.

Investigators who have Tier 2 access will see additional options (Figs. 3, 4) for downloading genetic and microbiome data, respectively. Files are broken down into pieces for ease of download, and investigators may elect to download all of a given data set, or only a subset of files to meet their research needs.

Investigators who have Tier 2 access will see this option for downloading genetic data. Files are broken down into pieces for ease of download, and investigators may elect to download all of a given data set, or only a subset of files to meet their research needs.

Investigators who have Tier 2 access will see this option for downloading genetic data. Files are broken down into pieces for ease of download, and investigators may elect to download all of a given data set, or only a subset of files to meet their research needs.

### Use cases

The Fox Insight data is particularly useful in studies of disease phenotyping, disease progression and risk factor analysis requiring large participant cohorts^15^. Examples of research projects that have used data from Fox Insight include a study of deep phenotyping for precision medicine^25^, a study of sex differences in PD presentation and progression^26^, and a study of risk factors such as coffee consumption and smoking^27^.

### Limitations

There are several limitations that researchers may want to consider when working with this data set^15^. Population bias is a potential issue as Fox Insight participants are self-selecting both at registration and at subsequent study visits. Approximately 90% of the participants in Fox Insight identify as being White, and 85% reside in the USA. Additionally, two-thirds of the participants reported having a yearly income of higher than $50,000. They also need to have access to, and be able to use, a suitable electronic data capture device such as a computer, tablet, or smartphone with an internet connection. Therefore, the Fox Insight cohort may not accurately represent the Parkinson’s Disease population at-large. We would suggest researchers interested in understanding underrepresentation carefully review demographic data.

For all participants, there may be reduced response rates over time in lieu of formal withdrawal from the study. Participation in the study is voluntary, and the average age of participants at enrollment is approximately 63 years, which may limit the generalizability of the findings to other age groups or populations. Moreover, participants with PD who regularly provide responses may be biased toward early and moderate stages of disease progression, because increased symptom severity may be detrimental to consistent participation. As a result, researchers interested in understanding or grouping participants by symptom severity may find Fox Insight to be a somewhat imbalanced sample, even though there are observations available for participants at all stages of PD progression. This should be taken into consideration when interpreting the findings of a given analysis, and caution should be exercised when generalizing to other populations or contexts.

Longitudinal data is not uniformly available for all participants^15^. Approximately one-third of participants either completed (1) only their baseline visit, or (2) only their baseline visit plus one or more one-time surveys. Proportionately, control group participants are less likely to have a high volume of longitudinal data available compared to participants with PD. Given the breadth of variables available and highly individual nature of participation patterns, researchers engaged in longitudinal analysis should carefully evaluate the availability of longitudinal data specific to a given research question, as study-wide longitudinal data may not provide an accurate picture^15^.

### Data quality

It is important to note that Fox Insight relies on data that is supplied directly by study participants, and this data is not independently validated. As some, but not all, fields have range checks or limits, it may therefore contain incorrectly entered numerical values (for example, biologically implausible height and weight values. As per the data use agreement completed by all users of the data, the data is provided on an “as-is” and “as available” basis. There are no guarantees of completeness or accuracy and researchers should take this into account when performing their analysis. Notwithstanding the above, a 2021 study of 203 Fox Insight participants found a very good level of agreement between self-reported diagnosis in Fox Insight and clinician-determined diagnosis^15^.

## Data Availability

Fox Insight was built by several technology partners, each with its own policies on code availability. Routine longitudinal assessments are developed through a web-based application built on Ruby on Rails® software by Mondo Robot and the code base is proprietary^28^. One-time questionnaires are deployed through Qualtrics®; while the survey platform code is proprietary, Qualtrics® provides an open-source application programming interface (API) for data processing. SQL code, developed at LONI, used to collate and process data is proprietary.

## References

[1] Marras C (2018). Prevalence of Parkinson’s disease across North America. NPJ Parkinson’s Dis..

[2] GBD 2016 Parkinson’s Disease Collaborators (2018). Global, regional, and national burden of Parkinson’s disease, 1990–2016: a systematic analysis for the Global Burden of Disease study 2016. Lancet Neurology.

[3] Schneider MG (2009). Minority enrolment in Parkinson’s disease clinical trials. Parkinsonism Relat. Disord..

[4] Moore SF, Guzman NV, Mason SL, Williams-Gray CH, Barker RA (2014). Which patients with Parkinson’s disease participate in clinical trials? One centre’s experiences with a new cell based therapy trial (TRANSEURO). J. Parkinsons Dis..

[5] National Institute of Health. NIH announces national enrollment date for All of Us Research Program to advance precision medicine. *National Institute of Health* https://www.nih.gov/news-events/news-releases/nih-announces-national-enrollment-date-all-us-research-program-advance-precision-medicine (2018).

[6] Musker M, Short C, Licinio J, Wong ML, Bidargaddi N (2019). Using behaviour change theory to inform an innovative digital recruitment strategy in a mental health research setting. J. Psychiatr. Res..

[7] Vogels, E. Millennials stand out for their technology use, but older generations also embrace digital life. *Pew Research Center.* https://www.pewresearch.org/fact-tank/2019/09/09/us-generations-technology-use/ (2019).

[8] Nielsen, Multifaceted connections. African-American media usage outpaces across platforms. *Nielsen.* https://www.nielsen.com/us/en/insights/article/2015/multifaceted-connections-african-american-media-usage-outpaces-across-platforms/ (2015).

[9] Perrin, A. Some digital divides persist between rural, urban and suburban America. *Pew Research Center.* https://www.pewresearch.org/fact-tank/2019/05/31/digital-gap-between-rural-and-nonrural-america-persists/ (2019).

[10] Faverio, M. Share of those 65 and older who are tech users has grown in the past decade. *Pew Research Center.* https://www.pewresearch.org/fact-tank/2022/01/13/share-of-those-65-and-older-who-are-tech-users-has-grown-in-the-past-decade/ (2022).

[11] Domingo A, Klein C (2018). Genetics of Parkinson disease. Handb. Clin. Neurol..

[12] Hernandez DG, Reed X, Singleton AB (2016). Genetics in Parkinson disease: Mendelian versus non-Mendelian inheritance. J. Neurochem..

[13] Nalls MA (2015). Diagnosis of Parkinson’s disease on the basis of clinical and genetic classification: a population-based modelling study. The Lancet Neurology.

[14] Winslow, A. R. *et al*. Self-report data as a tool for subtype identification in genetically-defined Parkinson’s disease. *Scientific Reports* **8** (2018). 10.1038/s41598-018-30843-6 PMC6113219 30154511

[15] Michael J (2019). Fox Foundation For Parkinson’s Research. Fox Insight Data Exploration Network (FoxDEN). University of Southern California Laboratory of Neuro Imaging..

[16] For an up-to-date copy of the Schedule of Activities – listing both longitudinal and cross-sectional surveys assigned by self-reported diagnosis – please visit: https://foxden.michaeljfox.org/insight/explore/insight.jsp.

[17] Kim HM (2018). Parkinsons Dis..

[18] Agile Alliance. What is Agile Software Development? Agile Alliance https://www.agilealliance.org/agile101/ (2019).

[19] Smolensky L (2020). Fox Insight collects online, longitudinal patient-reported outcomes and genetic data on Parkinson’s disease. Sci Data.

[20] Schneider, R. B. *et al*. Design of a virtual longitudinal observational study in Parkinson’s disease (AT-HOME PD). *National Library of Medicine* https://www.ncbi.nlm.nih.gov/pmc/articles/PMC7886038/ (2021).

[21] Schneider RB (2021). Parkinson Study Group AT-HOME PD Investigators. Design of a virtual longitudinal observational study in Parkinson’s disease (AT-HOME PD). Ann Clin Transl Neurol..

[22] For more information on SURE-PD 3, please visit: https://clinicaltrials.gov/ct2/show/NCT02642393. For more information on STEADY-PD III, please visit: https://classic.clinicaltrials.gov/ct2/show/NCT02168842.

[23] For more information on At-Home PD 2 (Grant ID: 1R01NS126933-01A1), please visit: https://reporter.nih.gov/search/GghuQ2d1aUWkoTfc9FG5rQ/project-details/10658165.

[24] Myers, T. L. *et al*. Video-based Parkinson’s disease assessments in a nationwide cohort of Fox Insight participants. *Clinical Parkinsonism & Related Disorders.* https://www.sciencedirect.com/science/article/pii/S2590112521000062 (2021). 10.1016/j.prdoa.2021.100094 PMC8299965 34316671

[25] Schalkamp AK, Rahman N, Monzón-Sandoval J, Sandor C (2022). Deep phenotyping for precision medicine in Parkinson’s disease. Disease Models & Mechanisms.

[26] Iwaki H (2021). Differences in the presentation and progression of Parkinson’s disease by sex. Movement Disorders.

[27] Gabbert C (2022). Coffee, smoking and aspirin are associated with age at onset in idiopathic Parkinson’s disease. J Neurol.

[28] *Ruby on Rails v5.2* (Ruby on Rails, 2018).

